# Supplementary material for: The interactome of intact mitochondria by cross-linking mass spectrometry provides evidence for coexisting respiratory supercomplexes
Source: Mol Cell Proteomics. 2017 Dec 8;17(2):216–32. doi: 10.1074/mcp.RA117.000470 (PMC5795388; doi:10.1074/mcp.RA117.000470)
Supplement: Supplemental Data [file supp_RA117.000470_133922_0_supp_23978_fzffwf.zip › spectra_annotation/mito_spectra_annotation/408-1-31-1-7-1.pdf]

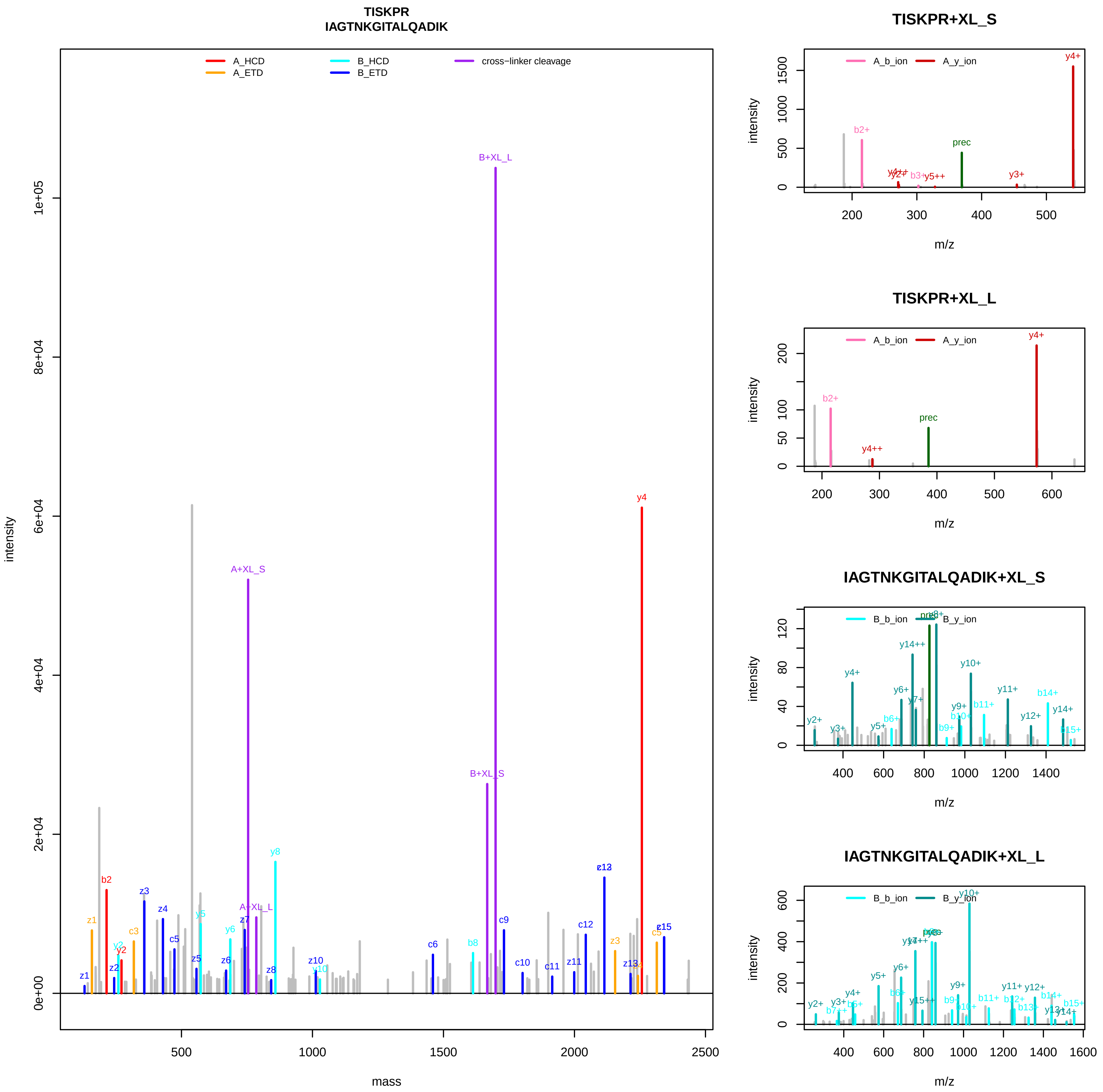
TISKPR
IAGTNKGITALQADIK
A_HCD
A_ETD
B_HCD
B_ETD
cross−linker cleavage
intensity
mass
0e+00
2e+04
4e+04
6e+04
8e+04
1e+05
500
1000
1500
2000
2500
B+XL_L
B+XL_S
A+XL_S
A+XL_L
y4
y8
b2
y2
z1
c3
z3
z4
c5
y5
z5
y6
z6
z7
z8
z10
y10
c6
b8
c9
c10
c11
z11
c12
z12
z13
c5
c15
z3
TISKPR+XL_S
TISKPR+XL_L
IAGTNKGITALQADIK+XL_S
IAGTNKGITALQADIK+XL_L
A_b_ion
A_y_ion
B_b_ion
B_y_ion
m/z
prec
b2+
y4+
y3+
y4++
y5++
b3+
y4+
y2+
y3+
y5+
y6+
y7+
y9+
y10+
y11+
y12+
y14+
y14++
b6+
b9+
b10+
b11+
b14+
b15+
y15++
b11+
b12+
b13+
y13+
